# Tumours of Nerve: An Electron Microscope Study

**DOI:** 10.1038/bjc.1962.52

**Published:** 1962-09

**Authors:** A. A. Barton

## Abstract

**Images:**


### TUMOURS OF NERVE: AN ELECTRON MICROSCOPE STUDY

A. A. BARTON

*From the Anatomy Department, Royal College of Surgeons of England, Lincoln's Inn Fields, London, W.C.2*

Received for publication May 19, 1962

IN mice after the repeated injection of dimethylbenzanthracene (DMBA) into the crushed sciatic nerve three kinds of malignant tumour have been found; those of the nerve or of the tissues immediately surrounding it, those of the epidermis, and those of mammary tissue.

Causey (1959) described the malignant tumours of mouse nerve produced experimentally by the injection of DMBA, including in his description a short account of the electron microscopic findings.

In the same year Woyke showed that similar tumours could be produced in both rat and rabbit (presented at a meeting of the Polish Anatomopathologist's Society, Poznan, in 1959—see Woyke, 1961). Both authors considered the possibility that some of the tumours were of Schwannian origin.

The ultrastructure of the tumours of the nerve itself is here considered in further detail and compared with that of proliferating Schwann cells (Barton, 1962) and extraneural fibrosarcomas, and with the mammary and basal cell carcinomas which occur at the site of operation.

### MATERIAL AND METHODS

The material used throughout the investigation was obtained from C+ strain virgin female mice. The sciatic nerve on the left side was exposed under ether anaesthesia and 9,10-dimethyl-1,2-benzanthracene (DMBA) (Grade C) dissolved in Tricaprylin injected in a mid-thigh position, so that a total of 0·01 mg. in 0·006 ml. was introduced. The nerve was gripped in smooth-ended forceps and clamped tightly by means of a pair of Spencer-Wells forceps so as to crush the nerve. This procedure was repeated at fortnightly intervals until a total of three injections had been given. Material for electron microscopy was fixed in ice-cold buffered isotonic 1 per cent osmic acid, washed in isotonic buffer solution and dehydrated in a graded water/ethanol series. It was stained with 1 per cent phosphotungstic acid in ethanol for two hours and embedded in Araldite. Sections were cut using a Cooke and Perkins ultramicrotome and examined in a Metropolitan Vickers E.M.6. electron microscope.

### RESULTS

### *Incidence of Tumours*

Fig. 1 is a graph constructed to show the incidence of the different types of tumour in those animals with a positive response to the application of carcinogen to the nerve.

It will be seen that tumours of nerve tend to occur maximally about five months after the first injection of carcinogen.

*Tumours of nerve*

Causey (1959), regarding the perineurium as the limiting structure of normal nerve, classified those tumours which disrupt it as intraneural and those which do not as extraneural. The same division has been adopted here. Dilatation of the nerve by the tumour mass, such as Causey (1959) described in mice and Woyke (1961) described in the rabbit, or continuity of nerve and tumour is a further macroscopic indication of intraneural origin. Extraneural tumours invade skin, muscle and bone, but do not infiltrate the nerve, which passes intact either through or alongside the tumour mass.

FIG. 1.—Time of incidence of palpable tumours in a series of 240 mice which showed a positive response to the injection of carcinogen in relation to the sciatic nerve. The time interval is calculated from the last injection of carcinogen.

—·—·—· Intraneural ——————— Extraneural
·········· Mammary - - - - - - - Skin

*Tumours of skin*

These are superficial tumours consisting either of well-differentiated cornifying squamous cell carcinomas or poorly differentiated basal cell carcinomas ; there are many types intermediate between the two, as discussed, for instance, by Willis (1953) ; they usually occur at the site of operation.

*Mammary tumours*

The number of mammary tumours recorded in Fig. 1 refers to the body as a whole ; there is no evidence to support the view that these tumours occur more frequently at the site of operation than elsewhere. Although the C+ strain of mice bears the milk factor which leads to the spontaneous production of mammary tumours there is no reason to suppose that the stimulus of repeated trauma affects the sites of their formation. They arise most frequently eleven months after the first injection of carcinogen.

### *Ultrastructure*

### *Tumours of nerve*

*Intraneural tumours.*—The following cell types occur in these tumours; elongated cells which form a closely packed tissue, such as is seen in Fig. 2, and cells with rounder outlines bound together to form a loose meshwork, as in Fig. 3. These configurations conform closely to the Type A and Type B benign Schwannomas described by Antoni (1920). The Type B arrangement grades into an epithelioid form where the cells may occur in the rosettes described by Causey (1959) and Woyke (1961).

*Type A tumours.*—It may be seen from Fig. 2 that the cells which form this type of tumour lie close to one another and in some areas regimentation in parallel array occurs, though the orderly arrangement of cells which characterises the fasciculated tissue such as is seen in a typical acoustic neuroma does not seem to occur. Cell boundaries are irregular, often forming elongated processes which lie parallel with one another, as in Fig. 4. In between the cells lies a tangled mass of collagen, finer fibrils and disrupted cells. It appears that this background of delicate fibres and intercellular material seen under the light microscope in benign (Willis, 1948) and malignant (Woyke, 1961) Schwannomas yields the altered staining reaction for collagen which forms a recognised feature of these tumours. Although patches of increased electron density may be seen at the cell margins of some cells in contact with one another, well-defined desmosomes, such as are seen in type B tissue, are rarely seen.

The cell cytoplasm is densely packed with granules of ribonucleoprotein. Mitochondria frequently occur, together with the vesicles and sinusoids of endoplasmic reticulum. The margins of the nucleus are irregular in outline and show a loss of parallelism between the inner and outer nuclear membranes. The latter

---

EXPLANATION OF PLATES

FIG. 2.—An intraneural tumour, Type A. The cells lie close to one another and possess irregular cell margins. Strands of collagen are seen at C. N = nucleoplasm. ×7000.

FIG. 3.—An intraneural tumour, Type B. In contrast to the previous figure the cells are loosely associated. Within the larger vesicles (V) lie evaginations of the cytoplasm cut in transverse section. ×10,000.

FIG. 4.—An intraneural tumour, Type A. The margin of this cell shows five elongated cell processes (P) lying parallel with one another. ×50,000.

FIG. 5.—An intraneural tumour, Type B. Desmosomes (D) are seen at the point of contact of two cells. N = nucleoplasm; C = collagen; CP = collagen precursor; ER = endoplasmic reticulum. ×20,000.

FIG. 6.—An intraneural tumour, Type B, showing a channel lined by collagen fibrils (C) and containing red blood corpuscles (RBC), a leucocyte (WBC) and cell processes (P). The tumour cells immediately adjacent to the channel are similar in appearance to those situated further away. ×5000.

FIG. 7.—An extraneural fibrosarcoma. Each cell is separate and surrounded by collagen fibres (C). Within the cytoplasm (Fig. 7a, ×32,000) are bundles of fibres similar in appearance to those seen in fibroblasts. ×16,000.

FIG. 8.—A basal cell carcinoma. The cell margin consists of fine cell processes with desmosomes (D) at many points. Within the cytoplasm (Fig. 8A, ×12,000) are bundles of tonofibrils (T). ×7000.

FIG. 9.—A mammary carcinoma. The cytoplasm contains A and B particles similar to those described in other virus induced tumours. At cell margin (CM) these particles are being extruded into the lumen. ×24,000.

2

3

4

5

6

7

8

9

Barton.

is often continuous with the vesicular structures in the cytoplasm, so that there is a continuity between the perinuclear space and these structures such as was described in secretory cells by Watson (1955). The nucleoplasm contains granules 100Å in diameter which may be aggregated to form clumps of electron-dense material, many of which lie along the nuclear margin.

*Type B tumours.*—It may be seen from Fig. 3 that the cell boundaries, although complex, present an over-all rounded outline ; the cytoplasm is limited by an electron-dense membrane approximately 200Å across. There are evaginations of the cytoplasm which form processes which may be several $\mu$ long. Some of these processes show invaginations which give rise to pockets of material within their own cytoplasm or one process may enfold another. It seems clear that some of the structures seen in the large vesicles in Fig. 3 are sections of these complexes cut transversely.

There is a generally loose association of the cells within the tumour mass. Whereas many are inter-related in the manner already described, many lie free, or with their margins touching. Fig. 5 shows two cells in which three of the points of contact are marked by diffuse areas of electron-dense material some 500Å wide, lying just beneath the cell wall of each. Between them there is a patchy increase in electron density, and the whole appearance recalls that of the desmosomes described in epithelial tissues by Fawcett (1958). Channels containing red blood corpuscles occur in the more densely packed parts of the tumour. There may be a rudimentary lining consisting of cell processes and strands of collagen, as in Fig. 6.

Within the spaces between the tumour cells lies a mass of debris consisting of granular material, isolated nuclei, and vesicles containing what appear to be the remains of mitochondrial membranes. The release of this material from cells with ruptured cell walls is seen in all parts of the tumour, and it is interesting to note that the nuclei and cytoplasm of such cells closely resemble those of cells near to blood vessels, where the supply of metabolites may be presumed to be adequate, so that the disintegration is more likely to be caused by a deficiency of the cell-wall than by a metabolic defect. Tangled masses of collagen fibres are also present in the intercellular spaces (Fig. 5) in addition to a closely associated fine fibrillary component which is probably a collagen precursor.

It may be seen from Fig. 3 that the cytoplasm is filled with rounded vesicles 0·1–1·0 $\mu$ in diameter, surrounded by a granular electron-dense material. Some of the vesicles contain cell-processes, as has already been shown, while others, usually the smaller ones, contain homogeneous material of varying electron density. Mitochondria with cristae mitochondriales are an unusual finding, and are randomly distributed throughout the cells which form the tumour mass. Particles 100Å in diameter are distributed throughout the cytoplasm, some are concentrated along the margins of elongated cisternae, forming the endoplasmic reticulum, while others occur singly, or concentrated into small groups.

Some cells contain the banded structures seen by Bellairs (1961) in degenerate cells of the chick embryo, as well as irregular droplets of electron-dense material. These are the only obvious signs of cytoplasmic pathology revealed by the electron microscope. The margins of the nucleus are irregular with a loss of parallelism between the inner and outer nuclear membranes as was seen in the Type A tissue, with evaginations of the outer membrane into the cytoplasm. The nucleoplasm is similar in appearance to that in Type A tissue, with coarse aggregations of ribonucleoprotein granules particularly noticeable along the margins of the nucleus.

*Extraneural tumours.*—Fig. 7 illustrates the features which characterise the tumours examined so far. It may be seen that the cell outlines are relatively simple and while fusiform variants do occur, they are usually short, scarcely ever reproducing the complexity of structure seen in the fibroblasts in developing tendon by Jackson (1955).

The cells are usually associated into tight groups, separated one from the other by collagen fibrils or fine filaments with banding which varies between 200 and 600Å. The cell walls are never clearly defined, presenting the appearance of fibroblasts described by Yardley (1960). Contacts between cells, with desmosomes, are scarcely ever seen. The cytoplasm contains filaments approximately 100Å in diameter, which fill the cell. These are often arranged in bundles which lack orientation. When cut transversely they appear as electron-dense circular profiles arranged in groups of fifty or so (Fig. 7a). The cell cytoplasm, although packed with these filaments, may contain a well-defined endoplasmic reticulum, with a regular arrangement of the ribonucleoprotein granules along its margins. Well-differentiated mitchondria are rare, but the cells contain rounded masses of electron-dense material 0·2 $\mu$ in diameter.

Nuclear outlines are irregular with a loss of the parallel arrangement of the two nuclear membranes. The nucleoplasm consists of granules 100Å in diameter, which are particularly evident at the nuclear margin.

### *Tumours of skin*

Under the light microscope these tumours show the great diversity of structure described by Zackheim, Simpson and Langs (1959). Using the electron microscope it may be seen that the individual cells are remarkably alike (Fig. 8). Each has a rounded profile with short extensions of the cell wall making the outline somewhat irregular and the nucleus usually reproduces this shape. The cytoplasm is filled with fine tonofibrils some 100Å in diameter and in some cells (Fig. 8A) these lie close to one another, lying in dense groups beside the nuclear or cell membranes. Some of the cells are stellate in outline with sheaves of tonofibrils in each extension, giving a typical prickle cell appearance as seen in the basal cells of normal skin. In many places well differentiated desmosomes lie at the point of contact of adjacent cells.

### *Mammary tumours*

These cells contain vacuoles (Fig. 9) surrounded by the characteristic A particles as described by Bernhard (1958) in other mammary tumours. All stages in the transformation of these particles into extracellular B particles are seen in this tissue, particularly at villous cell margins. Well marked desmosomes are present.

### DISCUSSION

The cell processes which form at the margins of many intraneural tumours are wrapped around one another or insinuated between other cells. In the case of loose associations such as were seen in the Type B cells, additional contacts are formed identical to the desmosomes of other epithelial tissues. This elaboration of cell surface is characteristic of both normal and proliferating Schwann cells which may enfold other Schwann cells or neuronal processes. In the case of myelinated nerve fibres, the cell continues to elaborate its surface in such a way that a coil is formed. Later, the myelin sheath is formed by the fusion of contiguous layers. The activity of the Schwann cell surface is especially evident in the case of damaged nerve, where a cell may exhibit phagocytic activity, engulfing the debris which may result from cell damage, or carbon black particles injected at the time of injury (Palmer, Rees and Weddel, 1961). When new nerve fibres start to form, the Schwann cells will engulf these, in spite of the fact that the Schwann cell still contains the remains of the old myelin sheath (Barton, 1962).

The cells of fibrosarcomas (extraneural tumours) possess a very simple arrangement of the cell surface and, in most cases, contact is prevented by the formation by the cells of large quantities of collagen or collagen precursor lying between them. While Schwann cells are also capable of forming collagen (Barton, 1962) they do so to a lesser extent, and in special circumstances ; for instance, when the cells have ceased the migratory movements which follow on axonal severance. The continued formation of collagen by fibrosarcoma cells and the failure to form secure contacts could provide an explanation for the failure of contact inhibition (Abercrombie, Heaysman and Karthauser, 1957), and account for their rapid spread in the body.

In spite of wide variation in the pattern of structure shown with the light microscope, the ultrastructural appearance of the cells which form these three classes of tumour arising in relation to the nerve is remarkably constant within each group. Many of the cells in mammary tumours contain virus particles ; the cells in skin tumours contain tonofibrils and granules and show desmosome contacts ; the cells of intraneural tumours possess elaborate cell surfaces, while fibrosarcomas, or extraneural tumours, have simple contacts separated by quantities of collagen.

The differences in structure and behaviour between cells of the intact prostatic acinus grown in whole organ culture and the cells migrating into the culture medium (Franks and Barton, 1960) suggests that the presence of spaces which afford opportunities for cell migration within tumour masses might account for the variation seen with the light microscope. It is of considerable interest that the cells of the intact prostatic acinus respond to testosterone while the migratory cells do not.

The cell walls of most tumour cells lack electron density, and basement membranes are often absent. Furthermore, specialization of the cell surface, as seen for instance in the formation of desmosomes, is minimal. However, a sufficient number of such structures is normally present to make identification of the tumour possible.

In some areas of the tumour where the cells lie in loose association with one another the plasmalemma may be lacking. The cell becomes disrupted and the nucleus and cytoplasm separated. It is thought that the appearance of these cells within the tumour mass is consistent with a defect of the cell surface and that while groups of cells are able to survive intact as a result of their mutual buttressing, in the case of isolated cells the fragility is such that the cell disintegrates. Certain chemotherapeutic agents may have a specific effect on the cell membrane and investigations into the structure of the cell surface of normal and malignant Schwann cells both before and after treatment with nitrogen mustard are in progress.

SUMMARY

A study has been made of the ultrastructure of the fibrosarcomas, Schwannomas, mammary and basal cell carcinomas which may arise in mice that have been injected with DMBA into the crushed sciatic nerve.

The cells of fibrosarcomas contain filaments 100Å in diameter within the cytoplasm. They have uncomplicated cell walls and are separated from one another by strands of collagen fibres.

Schwannomas have elaborated cell margins which show occasional desmosome contacts.

Mammary carcinomas reveal, in their cytoplasm, the presence of milk factor particles, while basal cell carcinomas contain tonofibrils and have complex cell margins. Both mammary and basal cell carcinomas form desmosome contacts.

The ultrastructural appearance of the cells of any one type of tumour is remarkably constant, in spite of the wide variation to be seen under the light microscope.

I gratefully acknowledge my indebtedness to Professor G. Causey for providing the material used in this investigation and for his constant help and advice. My thanks are also due to my wife, Mrs. Mary Barton, to Mr. S. A. Edwards, Miss Anne Broughton and to the staff of the photographic department for technical assistance.

Acknowledgments are due to the British Empire Cancer Campaign for financial assistance.

### REFERENCES

ABERCROMBIE, M., HEAYSMAN, J. E. M. AND KARTHAUSER, H. M.—(1957) *Exp. Cell Res.*, **13**, 276.

ANTONI, N. R. E.—(1920) ‘ Ueber Rückenmarkstumoren und Neurofibrome’. München and Wiesbaden (J. F. Bergmann).

BARTON, A. A.—(1962) *Brain* (in press).

BELLAIRS, R.—(1961) *J. Anat.*, **95** (1), 54.

BERNHARD, W.—(1958) *Cancer Res.*, **18**, 491.

CAUSEY, G.—(1959) *Acta Un. int. Cancr*, **15**, 142.

FAWCETT, D.—(1959) “ Structural specializations of the cell surface ”. In ‘ Frontiers in Cytology ’. Edited by Palay. New Haven (Yale University Press), pp. 19–41.

FRANKS, L. M. AND BARTON, A. A.—(1960) *Exp. Cell Res.*, **19**, 35.

JACKSON, S. F.—(1955) *Proc. Roy. Soc.*, B, **144**, 556.

PALMER, E., REES, R. J. W. AND WEDDELL, G.—(1961) *J. Anat. Lond.*, Supplement : ‘ Cytology of Nervous Tissue ’, p. 49.

WATSON, M. L.—(1955) *J. biophys. biochem. Cytol.*, **1**, 257.

WILLIS, R. A.—(1953) ‘ Pathology of Tumours ’. 2nd edition. London (Butterworth).

WOYKE, S.—(1961) *Cancer*, **14**, 1030.

YARDLEY, J. H. *et al.*—(1960) *Johns Hopk. Hosp. Bull.*, **106**, 381.

ZACKHEIM, H. S., SIMPSON, W. L. AND LANGS, L.—(1959) *J. invest. Derm.* **33**, 385.


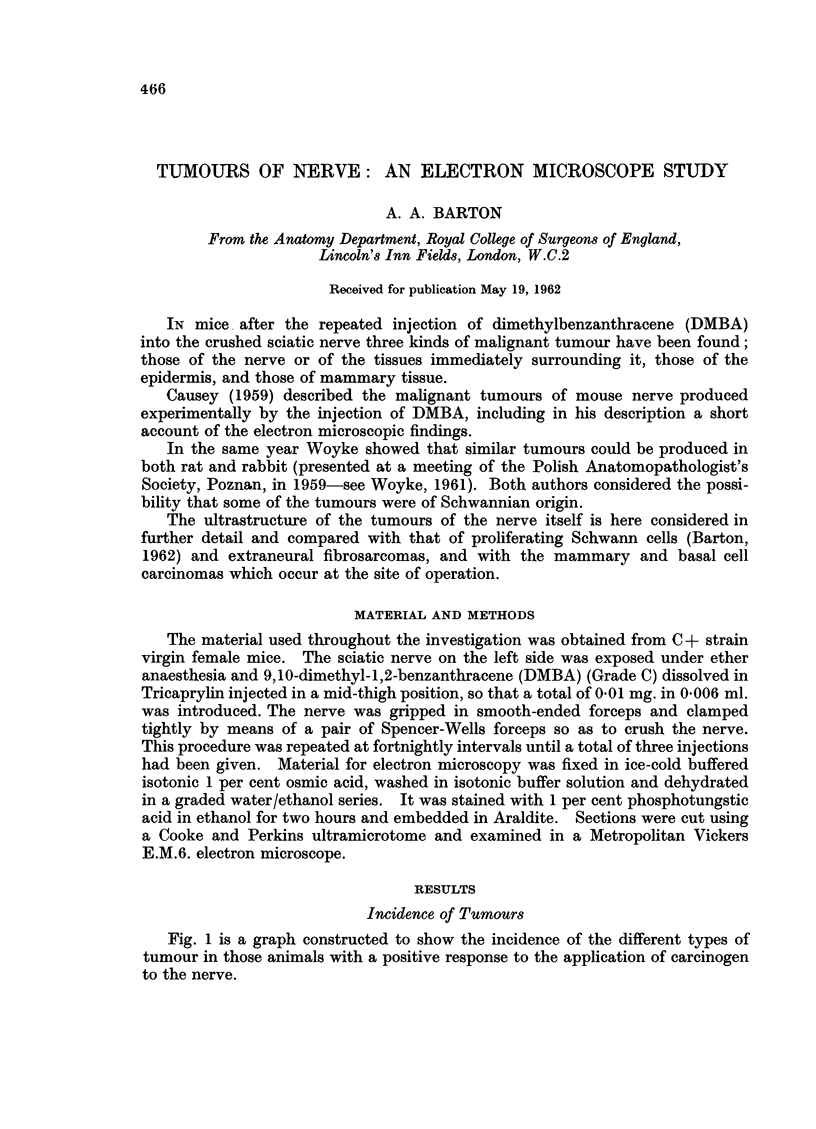

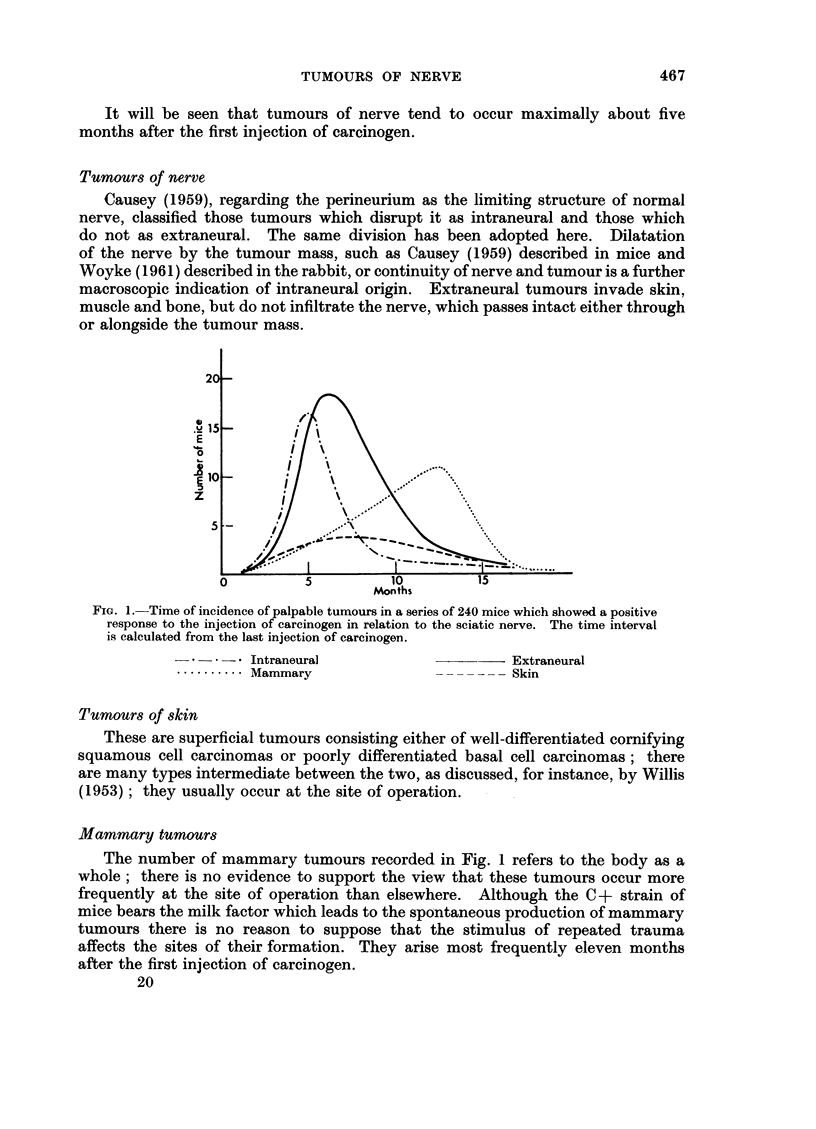

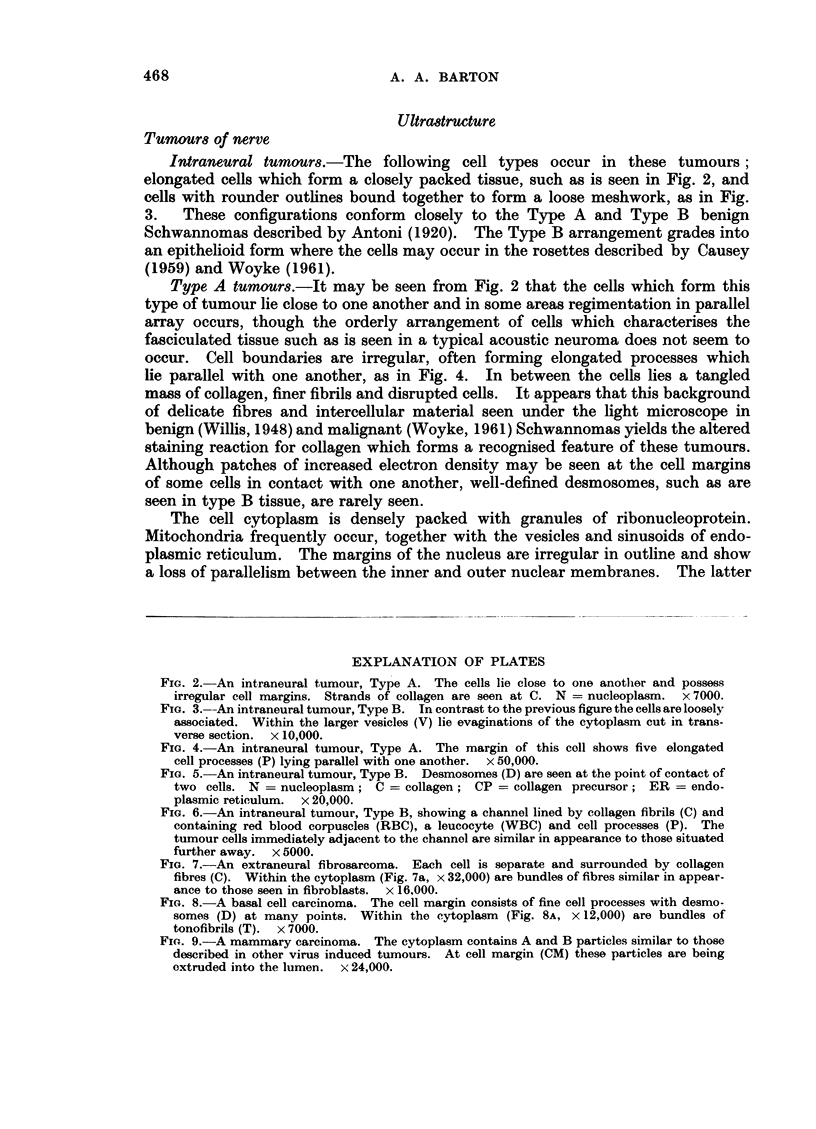

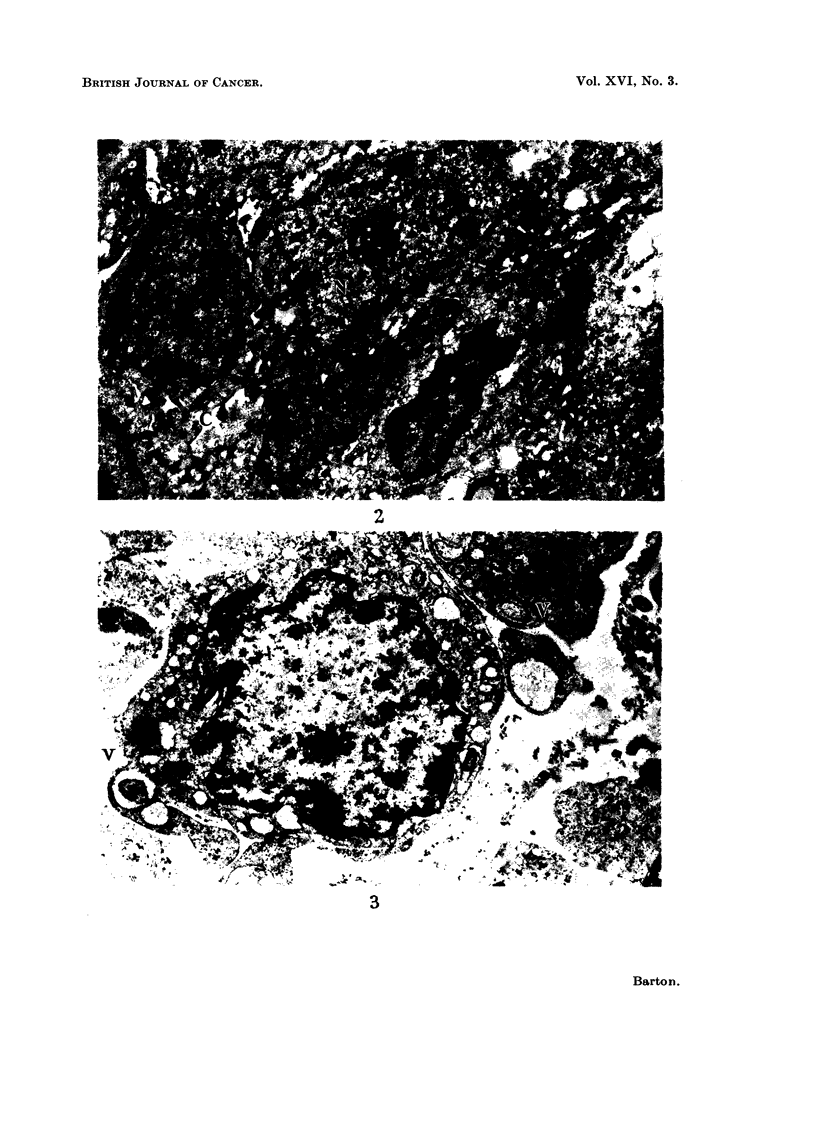

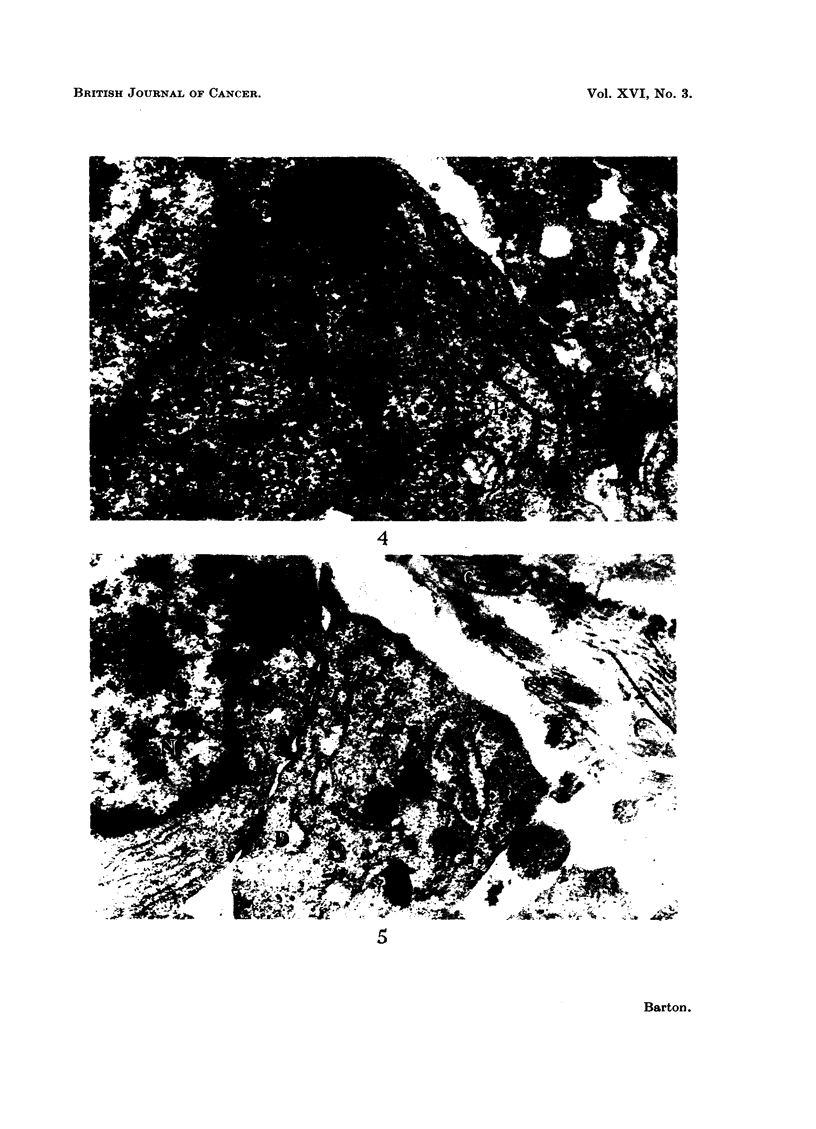

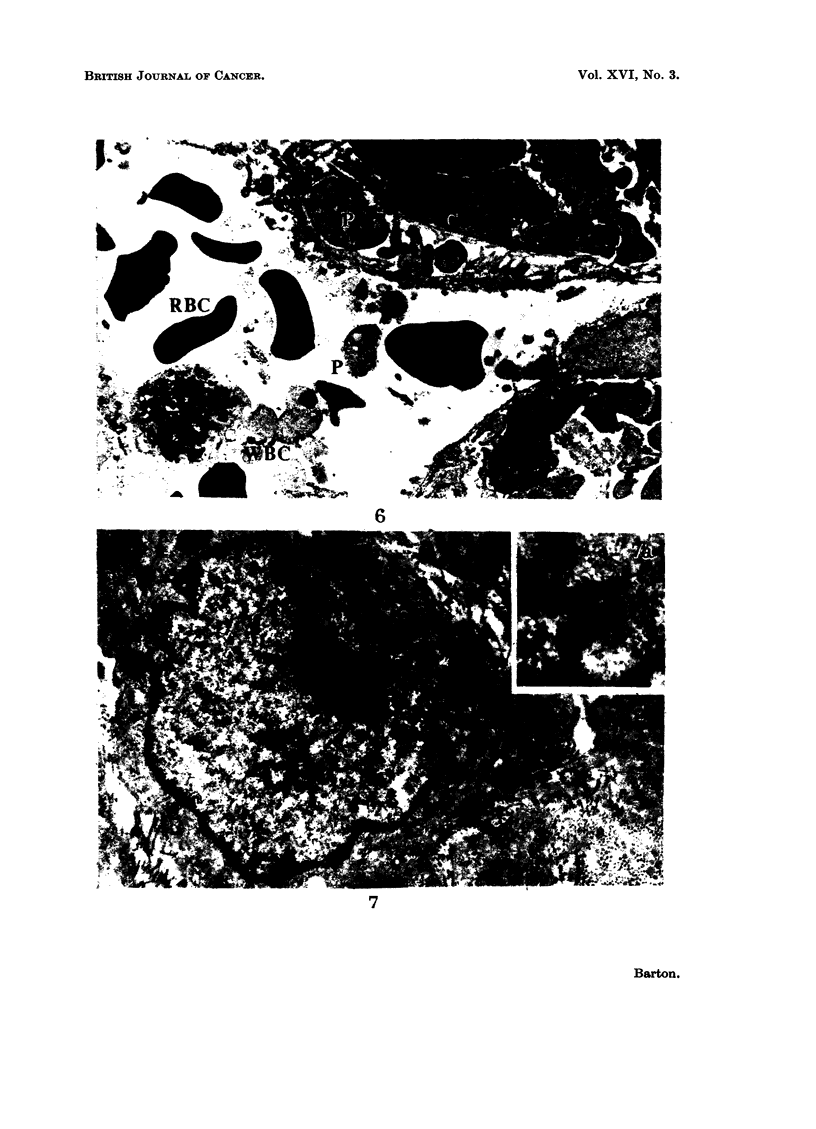

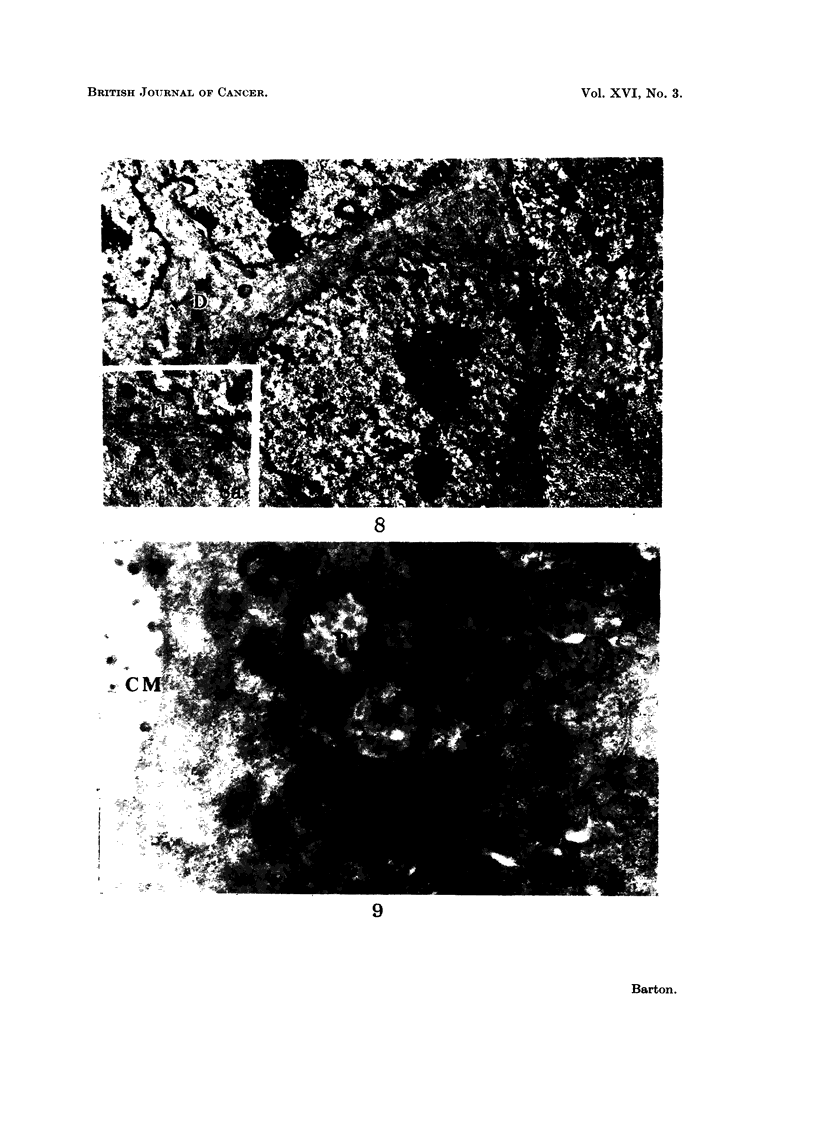

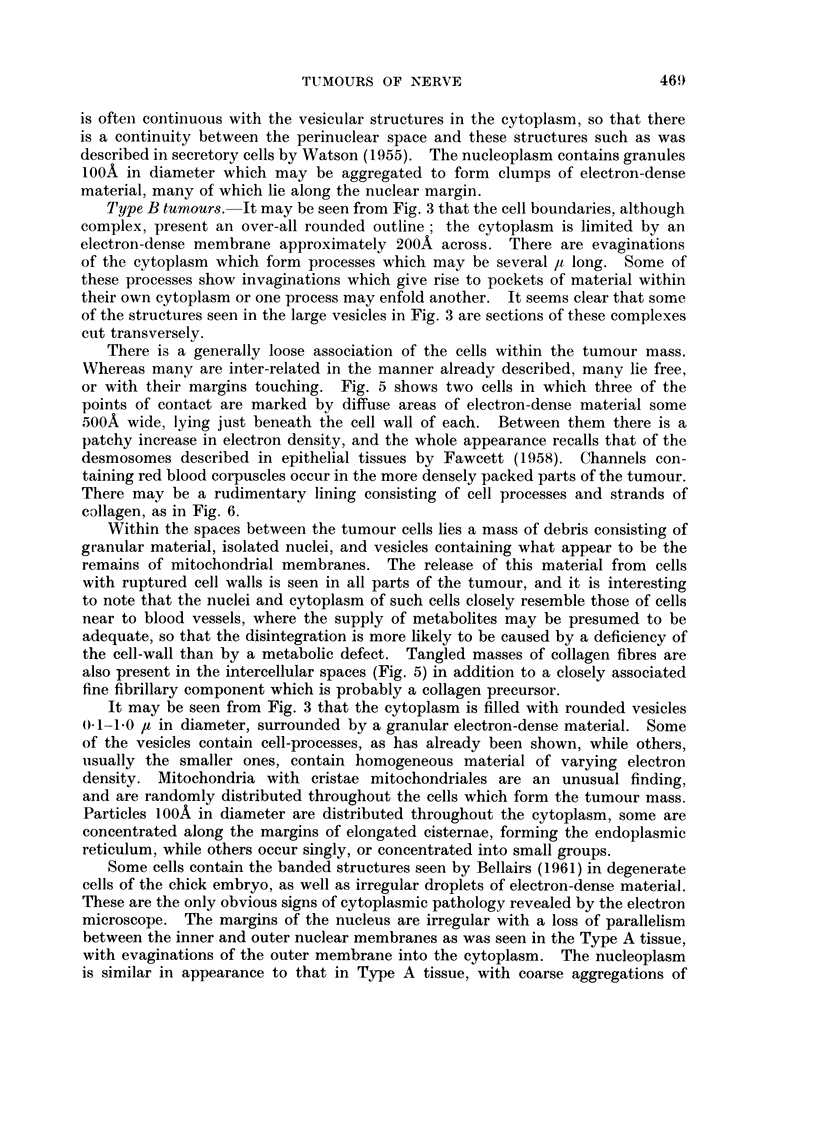

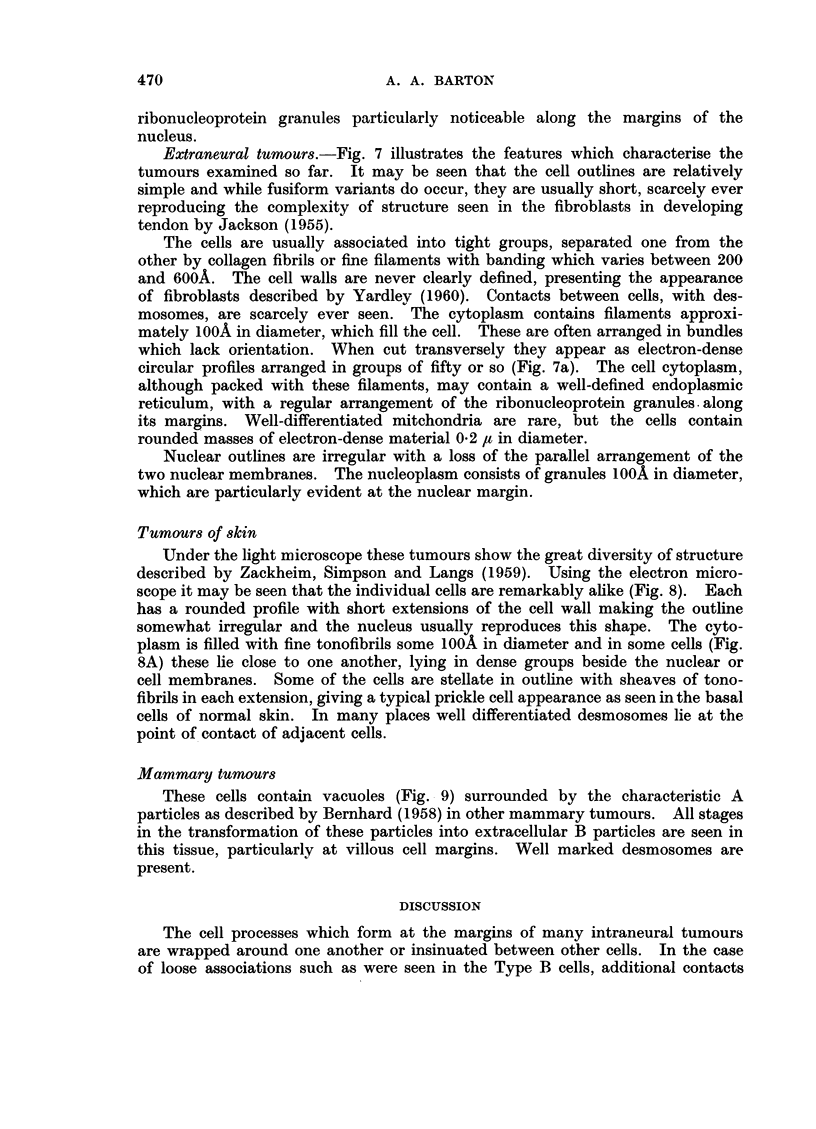

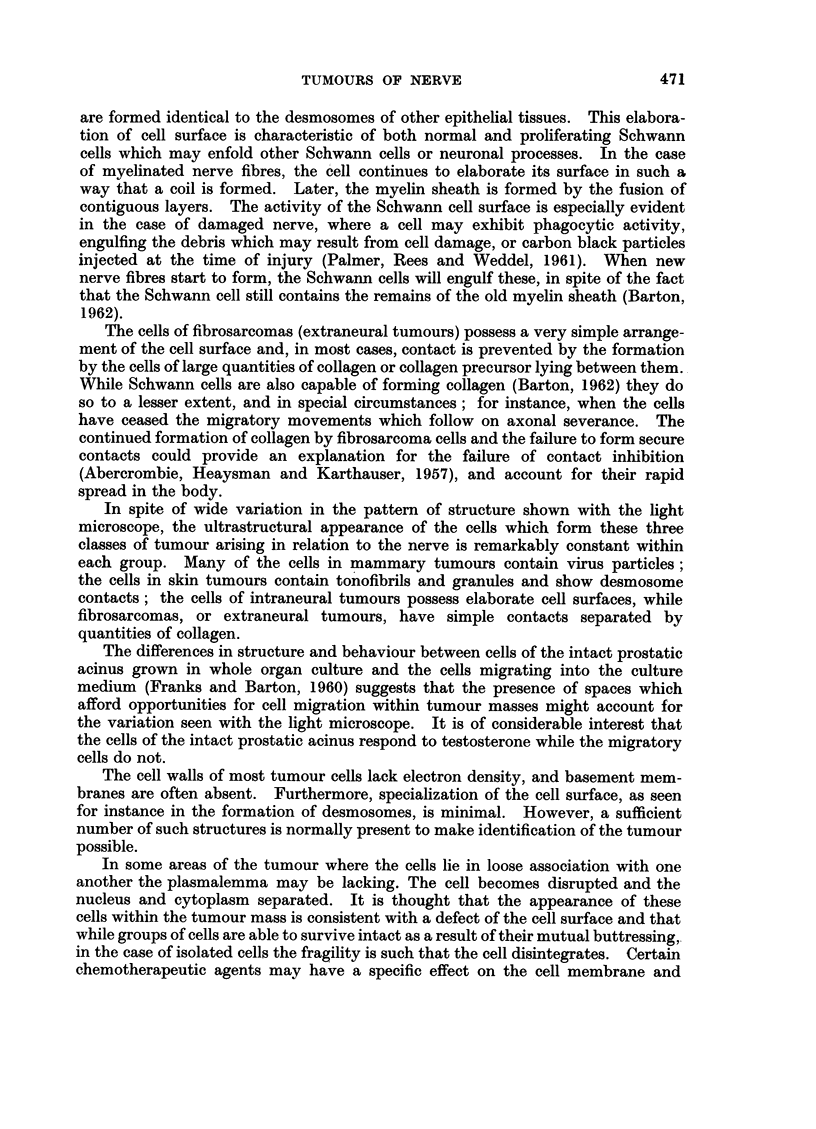

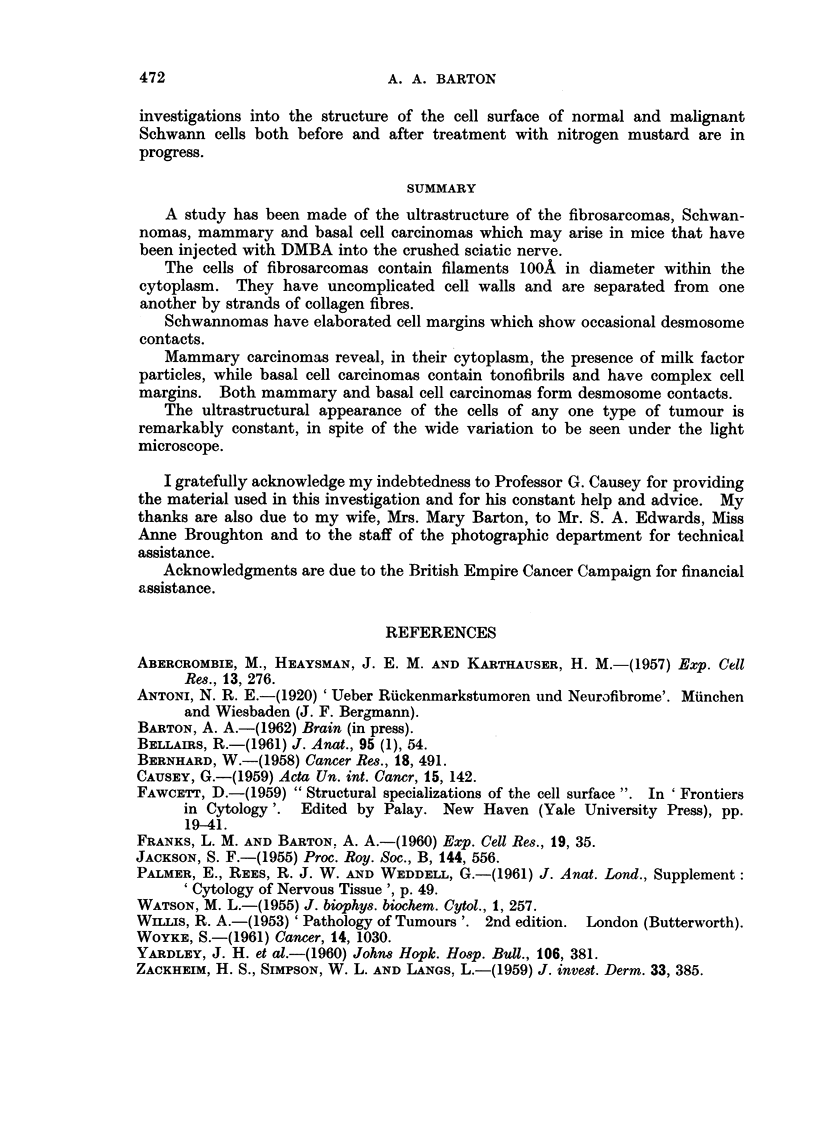


## References

[OCR_00466] ABERCROMBIE M., HEAYSMAN J. E., KARTHAUSER H. M. (1957). Social behaviour of cells in tissue culture. III. Mutual influence of sarcoma cells and fibroblasts.. Exp Cell Res.

[OCR_00475] BERNHARD W. (1958). Electron microscopy of tumor cells and tumor viruses; a review.. Cancer Res.

[OCR_00477] CAUSEY G. (1959). Experimental tumours of peripheral nerve in mice.. Acta Unio Int Contra Cancrum.

[OCR_00491] WATSON M. L. (1955). The nuclear envelope; its structure and relation to cytoplasmic membranes.. J Biophys Biochem Cytol.

[OCR_00494] WOYKE S. (1961). Experimental tumors of peripheral nerve trunks in rabbits.. Cancer.

[OCR_00496] YARDLEY J. H., HEATON M. W., GAINES L. M., SHULMAN L. E. (1960). Collagen formation by fibroblasts: preliminary electron microscopic observations using thin sections of tissue cultures.. Bull Johns Hopkins Hosp.

[OCR_00498] ZACKHEIM H. S., SIMPSON W. L., LANGS L. (1959). Basal cell epitheliomas and other skin tumors produced in rats and mice by anthramine and methycholanthrene.. J Invest Dermatol.

